# Supplementary material for: Pilot Testing of Useful Tools’ Validity for Frailty Assessment in Greece: Translated PRISMA-7 Tool, Modified Fried Criteria and Clinical Frailty Scale
Source: Healthcare (Basel). 2024 Apr 30;12(9):930. doi: 10.3390/healthcare12090930 (PMC11083930; doi:10.3390/healthcare12090930)
Supplement: Supplementary file 1 [file healthcare-12-00930-s001.zip › healthcare-2959513-Supplementary File S1 Prisma 7 Translation in Greek.pdf]

ΕΡΩΤΗΣΗ	ΑΠΑΝΤΗΣΗ	
Είστε 85 ετών ή μεγαλύτερος;	ΝΑΙ	ΟΧΙ
Είστε Άνδρας;	ΝΑΙ	ΟΧΙ
Σε γενικές γραμμές, έχετε κάποιο πρόβλημα υγείας λόγω του οποίου απαιτείται να περιορίσετε τις δραστηριότητες σας;	ΝΑΙ	ΟΧΙ
Χρειάζεστε τη βοήθεια άλλου ατόμου σε τακτική βάση;	ΝΑΙ	ΟΧΙ
Σε γενικές γραμμές, έχετε κάποιο πρόβλημα υγείας λόγω του οποίου απαιτείται να μείνετε στο σπίτι;	ΝΑΙ	ΟΧΙ
Αν χρειαστείτε βοήθεια, μπορείτε να υπολογίσετε σε κοντινό σας πρόσωπο;	ΝΑΙ	ΟΧΙ
Χρησιμοποιείτε τακτικά μπαστούνι, περιπατητήρα ή αναπηρικό αμαξίδιο για να μετακινείστε;	ΝΑΙ	ΟΧΙ

Centre d'expertise en santé de Sherbrooke. CESS©

• Raîche, M., Hébert, R., Dubois, M.F., Grégoire M, Bolduc J., Bureau C., Veil A. Le repérage des personnes âgées en perte d’autonomie modérée à grave avec le questionnaire PRISMA7 : développement, implantation et utilisation. La Revue de Gériatrie, 2007; 32(3): 209-218.

• Raîche M, Hébert R, Dubois M-F. PRISMA-7: A case-finding tool to identify older adults with moderate to severe disabilities. Archives of Gerontology and Geriatrics 2008; 47(1): 9-18.

Για τα Ελληνικά: Σούλης, Γ., Κυριακοπούλου, Ε.
