# Supplementary figures and images for: Pilot Testing of Useful Tools’ Validity for Frailty Assessment in Greece: Translated PRISMA-7 Tool, Modified Fried Criteria and Clinical Frailty Scale
Source: Healthcare (Basel). 2024 Apr 30;12(9):930. doi: 10.3390/healthcare12090930 (PMC11083930; doi:10.3390/healthcare12090930)

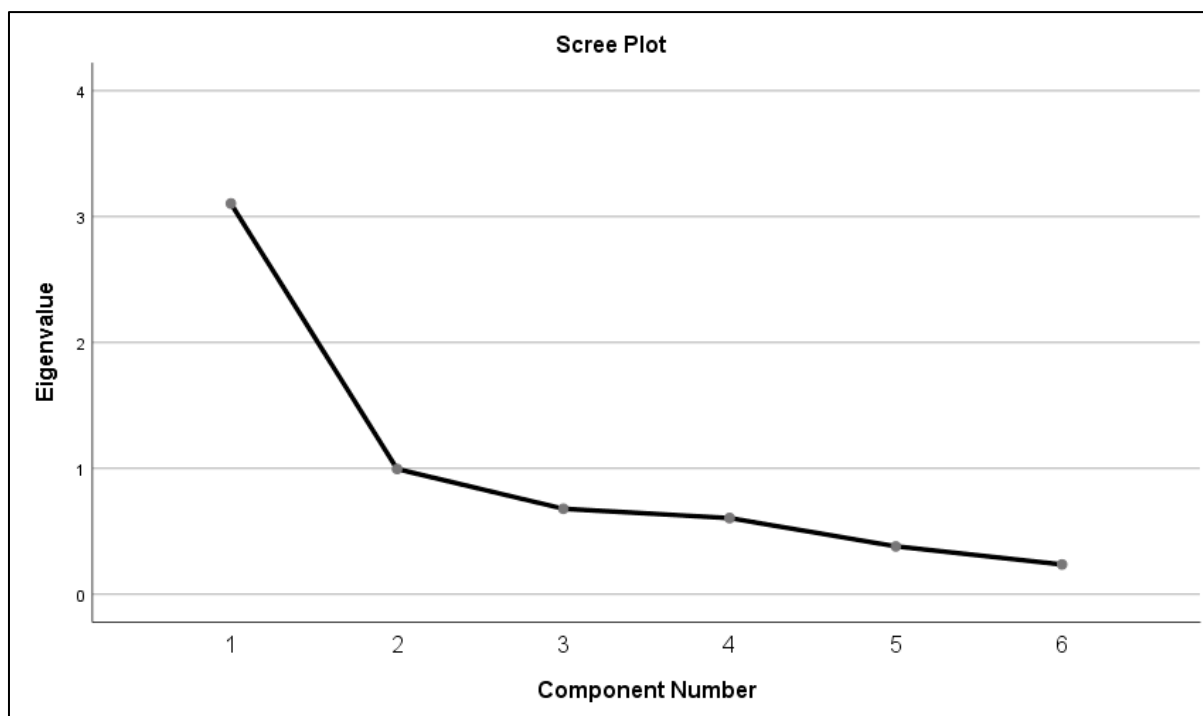

**Figure S2.** Scree plot.

Supplement: Supplementary file 1 [file healthcare-12-00930-s001.zip › healthcare-2959513-Supplementary Figure S1 Scree plot.pdf]
